# A systematic review on quality indicators for tight glycaemic control in critically ill patients: need for an unambiguous indicator reference subset

**DOI:** 10.1186/cc7114

**Published:** 2008-11-11

**Authors:** Saeid Eslami, Nicolette F de Keizer, Evert de Jonge, Marcus J Schultz, Ameen Abu-Hanna

**Affiliations:** 1 Department of Medical Informatics, Academic Medical Center, University of Amsterdam, Meibergdreef, 1105 AZ Amsterdam, The Netherlands; 2 Department of Intensive Care, Academic Medical Center, University of Amsterdam, Meibergdreef, 1105 AZ Amsterdam, The Netherlands

## Abstract

**Introduction:**

The objectives of this study were to systematically identify and summarize quality indicators of tight glycaemic control in critically ill patients, and to inspect the applicability of their definitions.

**Methods:**

We searched in MEDLINE^® ^for all studies evaluating a tight glycaemic control protocol and/or quality of glucose control that reported original data from a clinical trial or observational study on critically ill adult patients.

**Results:**

Forty-nine studies met the inclusion criteria; 30 different indicators were extracted and categorized into four nonorthogonal categories: blood glucose zones (for example, 'hypoglycaemia'); blood glucose levels (for example, 'mean blood glucose level'); time intervals (for example, 'time to occurrence of an event'); and protocol characteristics (for example, 'blood glucose sampling frequency'). Hypoglycaemia-related indicators were used in 43 out of 49 studies, acting as a proxy for safety, but they employed many different definitions. Blood glucose level summaries were used in 41 out of 49 studies, reported as means and/or medians during the study period or at a certain time point (for example, the morning blood glucose level or blood glucose level upon starting insulin therapy). Time spent in the predefined blood glucose level range, time needed to reach the defined blood glucose level target, hyperglycaemia-related indicators and protocol-related indicators were other frequently used indicators. Most indicators differ in their definitions even when they are meant to measure the same underlying concept. More importantly, many definitions are not precise, prohibiting their applicability and hence the reproducibility and comparability of research results.

**Conclusions:**

An unambiguous indicator reference subset is necessary. The result of this systematic review can be used as a starting point from which to develop a standard list of well defined indicators that are associated with clinical outcomes or that concur with clinicians' subjective views on the quality of the regulatory process.

## Introduction

Hyperglycaemia is frequently encountered in critically ill patients [1,2]. Even critically ill patients without diabetes develop hyperglycaemia. Until recently it was common practice to treat only marked hyperglycaemia in these patients, because hyperglycaemia was considered to be an adaptive response to critical illness [3]. Blood glucose control aiming to achieve normoglycaemia (blood glucose levels of 80 to 110 mg/dl), frequently referred to as 'tight glycaemic control' (TGC), decreases mortality and morbidity in critically ill patients [4,5]. It is the lowered blood glucose level (BGL) rather than the insulin dose that is related to reduced mortality and morbidity [6]. Attempts at achieving TGC, however, are not perfect and carry a risk for hypoglycaemia [4,5].

Several observational studies have reported on the quality of the glucose control process itself. The results and conclusions of these studies are contradictory [7]. Some show that the protocol prescribing the control process improves blood glucose control whereas others do not. Apart from differences in case-mix and in the associated therapy (for example, steroid therapy), two important issues hamper comparability between studies. The first impediment is the existing variability in the intervention's evaluation. The following interpretations, based on intention and on process, are both possible: the patient is intended to be treated according to a TGC protocol (for example, when a specific intensive care unit is designated an intervention group), independent of actual adherence to the glucose control protocol; or the characterization of the patient's blood glucose regulation is evaluated according to the actual intensity of blood glucose control. The latter interpretation requires agreement on the level of adherence to the TGC protocol in terms of timing of glucose measurements and insulin provision to qualify a patient as being on TGC. The second impediment concerns the variability in outcome measures; studies may not use a standard list of well defined indicators for evaluating the quality of glucose control. Work presented in this paper concerns this second impediment.

The objective of the present systematic review is to identify and summarize quality indicators for glucose control in published studies of TGC in critically ill patients. It also assesses the applicability of definitions of quality indicators and organizes the indicators into categories. This review may form a basis for future developments of a standard list of well defined indicators that may correlate with clinical outcomes or that reflect clinicians' intuition regarding the quality of a given regulatory process.

## Materials and methods

We searched for relevant English language articles based on keywords in title, abstract and MeSH terms, using Ovid MEDLINE^® ^and Ovid MEDLINE^® ^In-Process (1950 to 31 December 2007). The final literature search was performed on 31 December 2007.

The following search strategy was used to identify the relevant articles. In the first stage we searched for 'glucose' and 'insulin'. In the second stage we limited the search using 'critical illness', 'critical care' or 'intensive care'. The results of these two stages were combined using the Boolean operator 'and'. Searching was supplemented by scanning the bibliographies of the identified articles.

Two reviewers independently examined all titles and abstracts. Discrepancies between the two reviewers were resolved by consensus involving a third reviewer. Articles were selected if they reported original data from a clinical trial or observational study conducted in critically ill adult patients, and only if one of their main objectives concerned the evaluation of quality of TGC, with or without implementing an explicitly specified protocol. A study was defined as evaluating a TGC protocol if the (implicit or explicit) protocol implied an upper target range. Adherence to the protocol did not influence whether the study was included. Opinion papers, surveys and letters were excluded. Studies employing glucose-insulin-potassium protocols were excluded because they are not originally designed to achieve TGC.

From the selected papers, the same two reviewers extracted data on TGC quality indicators (their definition and applicability). A quality indicator was defined as a measurable quantity of the TGC process that may, alone or in combination with other quantities, indicate some aspect of its quality. This includes, for example, mean (or median) BGLs as well as interpretations thereof in terms of counts of hyperglycaemic events. Discrepancies between the two reviewers were again resolved by consensus after involving the same third reviewer. We then attempted to categorize the quality indicators into coherent categories that capture their essence.

## Results

Searching the online databases yielded 486 articles. Initial screening of titles and abstracts resulted in 50 articles eligible for further full-text review. One additional article was identified by reviewing bibliographies, for a total of 51 articles. Based on the full-text review, two studies were excluded because they turned out not to address original data, leaving 49 articles for detailed analysis. Only five out of 49 studies reported on a target upper limit above 150 mg/dl.

All quality indicators of the 49 studies are summarized in Tables 1 and 2.

**Table 1:** List of applied quality indicators

Indicator	Description	References
Hypoglycaemia^a^	Represented in different ways. In most studies it is represented as the number or percentage of measurements below a certain BGL value and/or number or percentage of patients who experienced at least one measurement below a predefined BGL value	43 articles [8-13,15-35,42-57]

BGLs over time	Represented as mean and/or median BGL values. Each BGL measurement or each patient was regarded the unit of observation. In one study, it was calculated as area under the glucose/time curve divided by total time per admission [10]. Mean or median BGL was also calculated: • at the end of IIT [52]; • after the target range was achieved [9,33,49,54]; • after stopping IIT [45,55]; • for patients who had admission BGL above a threshold [10]; • during IIT [27] or during the last 5 hours of IIT [57]; • after 24 hours [15]; or • 24 hours before, within and after IIT [39] or 48 hours after IIT [11]	37 articles [8-12,15,17,18,20-22,24,26,27,29,30,32-36,39,42-47,49-52,54-58]

Measurements in predefined BGL ranges	Represented as the number or percentage of measurements in a predefined BGL range: • during the study; • after the target is achieved [27,49,55,56]; or • at the start of IIT [21] Each BGL measurement, and in one study each patient [11], was considered the unit of observation	31 articles [9,11,12,17-21,25-28,30-33,35,36,42-45,47,49-51,55-57,59,60]

Time to capture defined BGL target	Represented as: • mean and median of time; or • by Kaplan-Meier curve, as in [17,20,23,25,49] In one study two successive BGL measurements in the target range were required before calculating time [20]	25 articles [8,9,11,15-17,20,21,23,25,27,29,30,32-34,43,45,46,49,52,53,55,56,59]

Frequency of measurements during the study	Represented as: • mean or median per patient [11,16,17,23-28]; • mean or median per patient treatment day or days [12,15,21,22,26,27,29-31]; • sampling interval (time) [13,31-36]; • median frequency per patient-hour [23]; • percentage of patients with more than one measurement in predefined time interval (every 2 hours, and so on) [31]; • frequency overall per day [12,21]; or • percentage of time in which at least one measurement per 2 hours was taken [32].	23 articles [11-13,15-17,20-36]

BGL at starting IIT	Represented as mean and median BGL	14 articles [9,11,13,16,21,27,29,33,46,52,54-56,59]

Protocol compliance	Compare measurement times suggested by protocol with actual times of measurements and/or pump speed during IIT and/or at the time of hypoglycaemic events [9,21]. In one studya time-motion method was used to measure discrepancy in timing of BGL measurements between protocol and actual [28]	13 articles [9,11,13,14,16,21,28-31,33,46,57]

Time in predefined range	Represented as: • mean of percentage of time per patient [12,32,58]; • median of fraction of time per patient [13,15,21,34]; • median of fraction of time per day [29]; • percentage of time for all patients [14,17,21,25]; or • percentage of time 24 hours before, within and after trial [39]	12 articles [12-15,17,21,25,29,32,34,39,58]

Hyperglycaemic events	Represented as: • percentage of time >180^b^, >250 (severe hyperglycaemia) [16], or between 151 and 200 and >200 (severe hyperglycaemia) [21]; • percentage of patients with at least one measurement per day ≥ 250 and ≥ 200) [22]; • percentage of measurements above 150 [11] or 180 [17-20]; or • percentage of measurements and patients with at least one BGL above the 180 level for more than 2 hours [13]	9 articles [11,13,16-22]

Morning BGLs	Represent as: • mean BGL around 06:00 hours [43], between 06:00 and 12:00 hours [50], or between 06:00 and 09:00 hours [19]; • mean lowest BGL between 06:00 and 09:00 [43]; • median between 06:00 and 08:00 hours [24] or between 03:00 and 06:00 hours [27]; or • mean of BGL, but morning time was not mentioned [20]	6 articles [19,20,24,27,43,50]

Hyperglycaemic index	Represented as median area between glucose-time curve and upper normal range divided by time per patient during the trial [15,24], in first 24 hours [23,25] or in first 48 hours [17]. Upper normal range was 207 [23], 117 [15], 108 [24], 120 [17], and 150 [25]. It was calculated with the same definition but without labeling as hypoglycaemic index [23,25]	5 articles [15,17,23-25]

Time until starting or adjusting IIT	Represented as mean or median of time until starting and/or adjusting IIT [9,10,27,28], or proportion of patients per time until starting IIT [12]. In one study a time-motion method was used [28]	5 articles [9,10,12,27,28]

Minimum and maximum recorded BGL	Represented as: • minimum and maximum recorded BGL over all patients [35,47]; or • median of minimum and maximum recorded BGL per patients [24] or per patient-day [29]	4 articles [24,29,35,47]

Number of patients with at least one BGL in predefined range	Represented as number and percentage per month [31] or at defined time interval after starting TGC [11] or during the study periods [60]	3 articles [11,31,60]

BGL change over time	Represented as: • speed of BGL change per hour [54]; or • BGL change in first 24 hours [15]	2 articles [15,54]

Number of patients who achieved or did not achieve target or predefined range	Represented as number and percentage	2 articles [17,27]

Number of positive culture	Represented as median (per patient) or rate (per year per patient).	2 article [31,59]

Target acquisition error	Represented as absolute value and percentage of difference between the target BGL and achieved BGL	2 articles [32,48]

^a^Hypoglycaemia is a concept in this table and related quality indicators are described in table 3. ^b^Unit of all BGL thresholds is mg/dl. BGL, blood glucose level; IIT, intensive insulin therapy; TGC, tight glycaemic control.

**Table 2:** List of applied quality indicators

Indicator	Description	References
Adequate daily blood glucose control	Represented as median hours spent each day within the target range per patient	1 article [21]

Correlation between within-run mean BGL and within-run mean coefficient of variance for hourly insulin rate	To illustrate whether hyperglycaemia after attaining target is correlated with variability in infusion rate	1 article [9]

Correlation between within-run mean BGL and within-run mean hourly insulin rate	To illustrate whether the protocol performed equally well independent of insulin resistance	1 article [9]

Distribution of the individual patient BGL mean in a predefined time interval	Represented in box plot	1 article [11]

Number of patients with well and poor BGL control	Represented as percentage of patients and defined as: • well controlled patient: <130^a ^BGL for more than half of the measured time; or • poor controlled patient: <130 BGL for less than or equal to half of the measured time.	1 article [31]

Odds ratio of achieving certain BGL	Per additional Intensive Care day and some drugs	1 article [50]

Number of patients having defined mean BGL	Represented as percentage of patients with mean BGL ≥ 200 for each day after surgery	1 article [22]

Probability density function for BGL measurements	Represented as a curve for comparison with other protocols	1 article [35]

Proportion of patient-day with mean BGL<200 and ≥ 200 and IIT at least in part of the day		1 article [22]

Number of report on necessary departure from protocol, clinical intervention or adverse events		1 article [35]

Time from admission to first BGL	Represented as mean of time [15]	1 articles [15]

Variability after achieving target	Represented as within-run mean (IIT episode) ± standard deviation and mean of within-run coefficient variance ± standard deviation (%)	1 article [9]

^a^Unit of all BGL thresholds is mg/dl. BGL, blood glucose level; IIT, intensive insulin therapy.

Most papers evaluated multiple quality indicators. The median number of quality indicators was five (range 2 to 10).

By inspecting the quality indicators, we arrived at four indicator categories based on the following: zones (adverse-zone [hypoglycaemia and hyperglycaemia] and in-range zone); BGLs (for example, mean morning BGL); time intervals (for example, time elapsed until an event occurs or time spent in some state); and protocol characteristics (for instance, blood sampling frequency).

The categories are not mutually exclusive. For example, the amount of time during which a patient is regarded to be in a hyperglycaemic state is related to an adverse-zone as well as to time. Below, we list indicators, in decreasing order of reported frequency, and describe our findings about them.

### Hypoglycaemia (adverse-zone and time categories)

Almost all studies reported at least one hypoglycaemia-related indicator (43/49 studies). Hypoglycaemia-related indicators address TGC safety. Because of its central position among the TGC quality indicators reported, hypoglycaemia is reported in Table 1 as an overall class of indicators.

Table 3 summarizes the concrete indicators used in this class along with their definitions.

**Table 3:** Hypoglycaemia quality indicator subgroups

Indicators	Description	Reference
Hypoglycaemic events	Reported thresholds^a ^for defining a BGL as hypoglycaemic event: • <40 [8,10,17-19,30,35,43]; • ≤ 40 [26]; • <45 [44,54]; • <50 [31,49,50]; • <54 [34]; • <57 [32]; • <60 [8,11,45,52,55]; • ≤ 60 [16,21]; • <63 [23]; • <65 [25]; • <70 [9,17,22,27,28,57]; • <72 [20]; or • threshold was not reported [48] Represented as: • percentage and number of measurements and/or patients with hypoglycaemic event during the trial, or normalized for duration of therapy [16]; • mean or median of events per patient-day [57]; • mean of events per patient [27]; • patients with at least one event per day [22]; or • in three studies, hypoglycaemic events did not occur and therefore were not reported [32,48,54]	31 articles [8-11,16-23,25-28,30-32,34,35,43-45,48-50,52,54,55,57]

Severe or marked hypoglycaemic events	Reported threshold for defining a BGL as severe hypoglycaemic event: • <40 [15,20,25,29,33,42,46,47,51,56]; • ≤ 40 [12,13,16]; • <48 [24]; or • in one study clinical finding defined as severe hypoglycaemia [8] Represented as: • percentage and number of measurements and/or patients with severe hypoglycaemic event; • mean or median of events per patient-day [29]; • In two studies severe hypoglycaemic events did not occur and therefore not reported [47,56]	15 articles [8,12,13,15,16,20,24,25,29,33,42,46,47,51,56]

Need for dextrose injection	Reported threshold for dextrose injection: • <45 [29]; • <54 [44]; • <60 [53]; • <63 [23]; • <65 [25]; or • threshold was not reported [21,56] Represented as: • percentage and number of patient with dextrose injection [21,23,29,44,53,56]; or • percentage and number of dextrose injections [25]	7 articles [21,23,25,29,44,53,56]

Mild or moderate hypoglycaemic events	Reported threshold for defining an BGL as severe hypoglycaemic event: • 40–59 [42]; • <60 [46]; • 40–60 [51]; • <63 [15] Represented as percentage and number of measurements and/or patients with a moderate hypoglycaemic event	4 articles [15,42,46,51]

Hypoglycaemia duration	Represented cumulatively [29], as median [24] and per patient [21]	3 articles [21,24,29]

Time until next in predefined range after hypoglycaemia	Represent as mean [8,49] or median [31] time	3 articles [8,31,49]

Duration of stopping IIT because of hypoglycaemia	Represented as median of percentage of time per patient	2 articles [29,33]

Next BGL after hypoglycaemia	Represented as mean BGL	2 articles [8,49]

Time until reaction to hypoglycaemic event	Represented as maximum time till hypoglycaemia recognition [8] or mean time till IIT adjustment after hypoglycaemic event [28]	2 articles [8,28]

Time from starting IIT until first hypoglycaemia	Represented as mean time	1 article [19]

Time till next BGL after hypoglycaemia	Represented as mean time	1 article [49]

^a^Unit of all BGL thresholds is mg/dl. BGL, blood glucose level; IIT, intensive insulin therapy.

In total, 15 different thresholds of BGL were used to define a hypoglycaemic event, varying from <40 mg/dl to <72 mg/dl. These included four different thresholds to define mild and moderate hypoglycaemia, and three different levels for defining severe or marked hypoglycaemia. Although a BGL <40 mg/dl was reported in eight studies as a hypoglycaemic event, 10 other studies considered this to be severe hypoglycaemia. One study reported severe hypoglycaemia only when a low BGL was accompanied by clinical symptoms such as sweating and decreased level of consciousness [8].

In some studies the number and/or percentage of BGL measurements below a given threshold and/or the number and percentage of patients with at least one measurement below this threshold were used as safety-related quality indicators. One article considered all measurements below the selected hypoglycaemic threshold value over a period of at least 1 hour to represent a single hypoglycaemic event; hence only when the BGL increased to within the normal range and then dropped below the hypoglycaemic threshold in a subsequent hour was it counted as a second hypoglycaemic event.

In other studies, the definition of hypoglycaemia was not clear, and it appeared that any measurement below the threshold was considered a hypoglycaemic event. Seven studies reported the number and/or percentage of dextrose injections when BGL was under a threshold value (using five different thresholds from 45 to 65 mg/dl) as quality indicators. Seven other indicators in this category were reported in at least one out of nine studies. Except for 'time from starting TGC till first hypoglycaemia', the other six indicators referred to the duration of hypoglycaemia or speed and quality of recovery after a hypoglycaemic event.

### BGL summaries over time (BGL category)

BGL summaries were used in 41 out of 49 studies. BGL summaries correspond to the efficiency of TGC. This indicator was calculated in different ways and was represented as mean and/or median. In some studies the BGL itself was the unit of observation. In other studies the mean BGL per patient or per time unit (for example, 1 hour) was the unit of observation. One study reported the mean and median of all BGLs as well as the mean and median of BGL during each intensive insulin therapy run [9]. 'Mean BGL' was also calculated as the area under the glucose/time curve divided by the total time per admission [10]. As long as there is no continuous measurement of BGLs over time (provided at any time point), this measure reflects mean BGL when BGL indeed behaves according to the assumptions underlying the interpolation of consequent BGL measurements.

BGLs are usually summarized as their mean at various points in time or in time intervals, and they are presented in graphs that show mean BGL versus time. Some studies used quality indicators that refer to the mean or median BGL measured at the end of TGC, before and after achieving the target range, or after stopping TGC. Summary of morning BGL was reported in six studies. The time used to define morning BGL varied among the studies. BGL at starting TGC (reported in 14 out of 49 studies) was another frequently used indicator in this group.

### Measurements and time in predefined BGL ranges (in-range zone and time categories)

**Figure 1:**
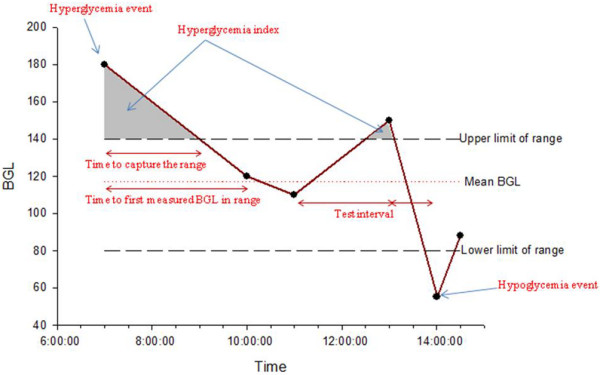
BGL measurements against time. Presented is a graph of hypothetical BGL measurements against time, showing hyperglycaemic and hypoglycaemic events, time to capture first (interpolated or measured) BGL in defined range, hyperglycaemic index and mean BGL.

Thirty-eight studies out of 49 examining the number of measurements and/or the time during which BGL was within a predefined range were reported. These indicators are intended to address TGC efficiency. In 31 out of 49 of these studies, the percentage of measurements within the predefined range was considered a proxy for the proportion of time in each predefined BGL range. In 12 out of 49 studies, the percentages of time during which BGL was within the predefined range were calculated, in most of them under the assumption that BGL was linear over time. As shown in Figure 1, under this assumption a straight line is drawn between each two consecutive BGL measurements, and the time to the intersection between the line and a threshold value defining the range was taken as the time spent within the predefined BGL range. Five studies used both the number/percentage of measurements within the predefined range as well as the time during which BGL was within the predefined range. The unit of observation differed also among these studies. Only in one study was the percentage of measurements within the predefined range calculated per patient, and the mean percentage per patient was reported [11].

### Time to capture the defined BGL target (time category)

The time needed to capture the defined BGL target was reported in 25 out of 49 studies and was represented as mean or median.

Similar to the time-related indicators in the predefined BGL range subcategory, in most of these studies it was unclear how this indicator was calculated. Linearity of BGL over time was explicitly mentioned in some studies [12-15] and appeared to have been assumed in other studies. It is possible that some of the studies might have used the time needed to capture the actual first BGL measurement within the target range, instead of the interpolated value shown in Figure 1.

### Hyperglycaemic indicators (adverse zone category)

Although the reduction in duration of a hyperglycaemic period forms a major goal of TGC, only 13 out of 49 studies explicitly mentioned how a hyperglycaemic event or indicator were defined. The threshold for considering a BGL measurement to be hyperglycaemic varied among studies from >150 to >250 mg/dl. Four different thresholds for hyperglycaemia and two thresholds for severe hyperglycaemia were reported. Six studies considered a BGL >180 mg/dl to be hyperglycaemic. Among the definitions, hyperglycaemia was identified as the percentage of time or of measurements above the threshold by seven studies [11,16-21], by one study [22] as the percentage of patients with at least one measurement above the threshold per day, and by one study [13] as a BGL above the threshold for at least 2 hours.

The hyperglycaemic index of BGL was defined by determining the area under the curve of BGL over time that is above the hyperglycaemic threshold divided by time per patient [15,17,23-25]. The thresholds varied between these studies (from 108 to 207 mg/dl).

### Sampling of BGL during the study, BGL at starting TGC and adherence to protocol (protocol category)

Sampling of BGL during the study was represented as mean or median of number of measurements per patient [11,16,17,23-28], per patient treatment day [12,15,21,22,26,29,30] or over 2 days [31], sampling interval [13,31-36], frequency per patient hour [23], percentage of patients with more than one measurement in a predefined time interval (2 hours) [31], or overall per day [12,21], and as the percentage of time during which at least one measurement per 2 hours was taken [32].

Frequent BGL measurement is a key element in TGC, in order to steer the process in a timely manner. However, greater sampling frequency increases nursing and laboratory utilization [30]. In some studies [14,18,19] the total number of BGL measurements was reported.

Adherence to protocol (reported in 12/49 studies) is another frequently used indicator. Evaluation of adherence to protocol mainly focused on the difference between the protocol-recommended time of the next BGL test and the actual time of testing.

The remaining indicators (19/30) were mentioned in fewer than six studies, and 12 of them in only one study.

On the whole, the included studies did not comment on why a specific group of indicators was selected, and – after further inspection – we could not find an association between indicator selection and patient population, disease or specification of the designed protocols.

## Discussion

We have identified, listed and categorized TGC quality indicators, as used in 49 studies. In our search for studies pertaining to TGC, we allowed any synonym, without limiting the search *a priori*. A limitation of our search is that we addressed only studies in which evaluation and quality measurement formed a main objective; we might therefore have missed some studies with a limited evaluation and quality measurement focus. In addition, frequency was used as the ordering principle for presenting and describing indicators. Although this approach provides a good overview of the popular indicators used, it may overlook less frequent but useful indicators. Finally, although indicator categories are useful in terms of managing and understanding indicators, their induction is subjective. One may for example also consider the complexity of calculation of indicators (for instance, calculating mean BGL is simpler and faster than time-weighted mean BGL).

To our knowledge this is the first review dedicated exclusively to quality indicators for TGC in critically ill patients. Existing reviews on TGC have focused on its effects [7,37]; evidence of its utility and its advantages were reported, and ways to implement TGC protocols successfully discussed.

Indicators and indicator groups have merits and limitations. Measures of mean BGL may mask measurements within adverse zones (for instance, two high BGLs may 'compensate' for one or more BGLs that are too low). Looking at hypoglycaemia and hyperglycaemic events separately would solve this problem, but this requires a way to combine both indicators into one quality indicator of blood glucose management. The Glycaemic Penalty Index, proposed very recently [38], is an attempt to address each zone and combine the two results. Indicators that neglect measurement timing, including the Glycaemic Penalty Index, may be sensitive to sampling. For example, the mean BGL of two determinations yielding the same BGL value taken at t_1 _and t_2 _or at t_1 _and t_3_, where say t_3 _> t_2_, will provide the same result, although the BGL – behaving as a function of time – may differ markedly. The hyperglycaemia index, which measures the area under the BGL over the time during which it was above a threshold, can mitigate this problem. The use of BGL measurement as the independent unit of observation neglects the within-patient correlation in BGLs. On the other hand, when providing summaries at the patient level, some information is lost. Finally, a statistical point worth noting is that most BGL distributions are log-normal rather than normal [39], and hence nonparametric measures such as the median and interquartile range are likely to be more appropriate for summarizing the data and inference [40].

Because hypoglycaemia is the main potential risk from implementation of a TGC protocol, almost all studies reported at least one indicator related to hypoglycaemia. The number of hypoglycaemic events before and after TGC implementation and/or the management of these events form the main TGC safety indicators. However, we found several definitions and ambiguous terminology for defining a blood glucose measurement (or a set of measurements) a hypoglycaemic event. Hypoglycaemic events were usually represented as the percentage or number of measurements below a defined level. Based on most glucose management protocols, the next BGL measurement after a hypoglycaemic event should be taken within 15 to 30 minutes. Only one study clearly stated that all measurements below the hypoglycaemia threshold over 1 hour after the first hypoglycaemic measurement were considered part of a single hypoglycaemic event or episode. In other studies it was not clear how these hypoglycaemic measurements where dealt with within a short interval, and hence whether they were regarded a single or as multiple events. Some studies also reported the number of dextrose injections to address this problem, where each injection corresponds to one hypoglycaemic event regardless of the number of measurements within the short interval. Even in these studies, the criteria and the BGL threshold for dextrose injection were different.

Indicators such as the percentage or number of BGL measurements and the time during which BGL was within predefined ranges were frequently used to represent the time duration in each predefined BGL range. However, the predefined ranges were different in the various studies, once again hampering comparability among them. Summary measures themselves, like mean and median of BGLs, were calculated with different units of analysis. Studies reporting the percentage of measurements tended to base calculations on all BGL measurements, regardless of the number of measurements of per patient.

In contrast, the percentage of time was calculated by taking a summary of each patient as the unit of analysis. These two ways of performing calculations do not necessarily yield the same results, because of within-patient correlations in measurements. It seems prudent to provide both results.

The strong relation between hyperglycaemia and mortality and morbidity is well known from the literature. Hence, hyperglycaemia reduction forms the main goal of TGC. Surprisingly, only nine studies explicitly defined a hyperglycaemic event and employed different definitions of an event in terms of timing and the BGL thresholds (between 150 to 250 mg/dl). Reporting the fraction of time above a threshold instead of the percentage of measurements above a threshold, without time consideration, seems more useful as a proxy for reducing time in a hyperglycaemic state. The hyperglycaemia index – calculated as the area between the curve and the hyperglycaemia threshold divided by time – seems to be a useful time-weighted indicator for hyperglycaemia. In some other studies, the percentage of BGL measurements or time in a predefined BGL range above the defined normoglycaemic threshold was reported but without explicitly labeling them as hyperglycaemia.

The quality of TGC in individual patients was rarely reported. Useful indicators include the percentages of patients with well and poorly controlled BGL, as defined by Carr and coworkers [31]; patients with at least one BGL outside the blood glucose target range; and patients who were not within the target blood glucose range

On the whole, the authors of studies did not explain their choices of specific subsets of indicators. It is conceivable that an indicator was described by a specific statistic such as a median because of an underlying non-normal distribution, in order to permit sound statistical inference. Although this may explain the specific choice of a statistic, it does not account for the choice for the underlying concept in the first place.

## Conclusion

When comparing the results of studies, one must consider differences in case-mix, in insulin therapy, in other associated therapies, in the power of the analysis and in outcome measures. The latter was the focus of this paper. The ambiguity and variability in the definitions of indicators and the threshold values for reporting an event as hypoglycaemia or hyperglycaemia severely hamper comparability among studies. A main problem is the absence of a 'gold standard' against which to compare indicators. Although there are almost no studies comparing different glycaemic metrics with relevant clinical outcomes, such as severity-associated mortality, deciding upon a common glycaemic vocabulary is an essential first step.

One possible useful way to proceed is to investigate further the relationship between indicators and clinical outcomes, for example their prognostic value (for example [24,41]). A second possible way is to ask a committee of experts to assess, for a wide range of patients, the perceived adequacy of TGC. Ideally, for this sample of patients the BGL would be continuously measured, with insulin provision being based only on protocol-based measurement sampling. Because this is an ethically questionable approach (because not all measured BGLs are acted upon), an alternative is to attempt to achieve very high sampling of BGL measurements. Then, the indicators could be assessed according to their concordance with how well BGL is controlled, as assessed by expert opinion. This approach is subjective but it can provide important insight into the merits of indicators. In the meantime, studies should report on a more comprehensive set of indicators, including at least one pertaining to each of time, hyperglycaemia and hypoglycaemia. One should also report results at the measurement as well as patient level. An important message of this review is that many indicators are not but should be precisely defined, using formulas when necessary, to facilitate their assessment and comparability.

## Key messages

• TGC indicators differ widely in their definitions, even when they are meant to measure the same underlying concept.

• Many definitions of indicators are not precise, limiting their applicability and hence the reproducibility and comparability of research findings.

• An unambiguous indicator reference subset is necessary for evaluating quality of TGC.

• The result of this systematic review can be used as a starting point from which to develop a standard list of well defined indicators, which are associated with clinical outcomes or concur with clinicians' subjective views on the quality of the regulatory process.

## Abbreviations

BGL: blood glucose level; TGC: tight glycaemic control.

## Competing interests

The authors declare that they have no competing interests.

## Authors' contributions

All authors made substantial contributions to the study design and methods. SE, AA and NdK performed the literature search, evaluated studies, extracted data, analyzed data and drafted the manuscript. All authors interpreted the results and were involved in revising the final manuscript.
